# Rediscovering the classic osteopathic literature to advance contemporary patient-oriented research: A new look at diabetes mellitus

**DOI:** 10.1186/1750-4732-2-9

**Published:** 2008-07-21

**Authors:** John C Licciardone

**Affiliations:** 1 The Osteopathic Research Center, University of North Texas Health Science Center, Fort Worth, TX, USA

## Abstract

Patient care experiences represent opportunities for establishing theories, testable hypotheses, and data to assess the potential use of osteopathic manipulative treatment in various disease conditions. The re-analysis of Bandeen's 1949 raw data described herein summarizes the effects of osteopathic manipulative treatment involving pancreatic stimulatory and inhibitory techniques in diabetic and non-diabetic patients seen over a 25-year period of clinical practice. Bandeen's data demonstrate a reduction in blood glucose levels at 30 and 60 minutes following pancreatic stimulation in 150 diabetic patients, and an elevation in blood glucose levels at 30 and 60 minutes following pancreatic inhibition in 40 non-diabetic patients. Such patient-oriented research conducted during the classic era of osteopathy in the United States provides a foundation and data for generating hypotheses about the potential mechanisms of action of osteopathic manipulative treatment. Osteopathic investigators would be well-served to rediscover the classic osteopathic literature to help advance contemporary evidence-based medicine.

## Rediscovering the classic osteopathic literature

The transformation from osteopathy to osteopathic medicine that occurred during the twentieth century in the United States [1] increased the scope of practice and patient care responsibilities of osteopathic physicians. However, this evolution from a classic focus on osteopathic manipulative treatment (OMT) to "full-service healthcare" [2] may have shifted emphasis away from patient-oriented research uniquely related to osteopathic principles. The National Institutes of Health defines patient-oriented research as that conducted with human subjects (or on material of human origin such as tissues, specimens, and cognitive phenomena) for which an investigator directly interacts with human subjects, including studies of mechanisms of human disease, therapeutic interventions, clinical trials, and the development of new technologies [3].

Patient care experiences represent opportunities for establishing theories, testable hypotheses, and, in some cases, data to assess the potential use of OMT in various disease conditions, including those not considered to be musculoskeletal disorders. Two such examples of patient-oriented research conducted during the classic period of osteopathy in the United States involve OMT in stimulating immunity [4,5], and in treating pandemic influenza [6]. In both cases, contemporary investigators have rediscovered these classic studies in efforts to advance the osteopathic research agenda.

Raw data from within-subjects observations conducted by Castlio and Ferris-Swift during the 1930s involving the use of splenic pump manipulation to stimulate the immune system in persons without acute infections [4] and in hospitalized patients [5] were recently re-analyzed using modern statistical techniques [7,8]. Despite limitations in research methodology and data collection common to the classic era, these re-analyses found that there was some empirical basis to support the hypothesized benefits of splenic pump manipulation in stimulating immunity. Similarly, data gathered during the 1918–1919 pandemic [6] have bolstered osteopathic principles and mechanistic theories in support of using OMT in patients with influenza [9]. In fact, a comprehensive series of OMT techniques was recently described and illustrated as potential treatment in the event of a pandemic of avian influenza or "bird flu" [9], although others have called for more rigorous research, intensive planning, and coordinated medical and public health responses prior to the potential implementation of such interventions on a large scale [10].

## A new look at Bandeen's classic osteopathic research on diabetes mellitus

Osteopathic physicians commonly enter primary care specialties [11] and thus often have the opportunity to use OMT in a variety of disorders, including both musculoskeletal and non-musculoskeletal conditions. Diabetes mellitus is one such disorder commonly managed by osteopathic physicians; however, research that addresses osteopathic palpatory findings or OMT in patients with diabetes mellitus is limited. Two series of within-subjects observations made by Bandeen in his clinical practice during the classic era of osteopathy, however, provide some insight into the potential benefits of OMT in complementing the management of patients with diabetes mellitus [12].

**Figure 1:**
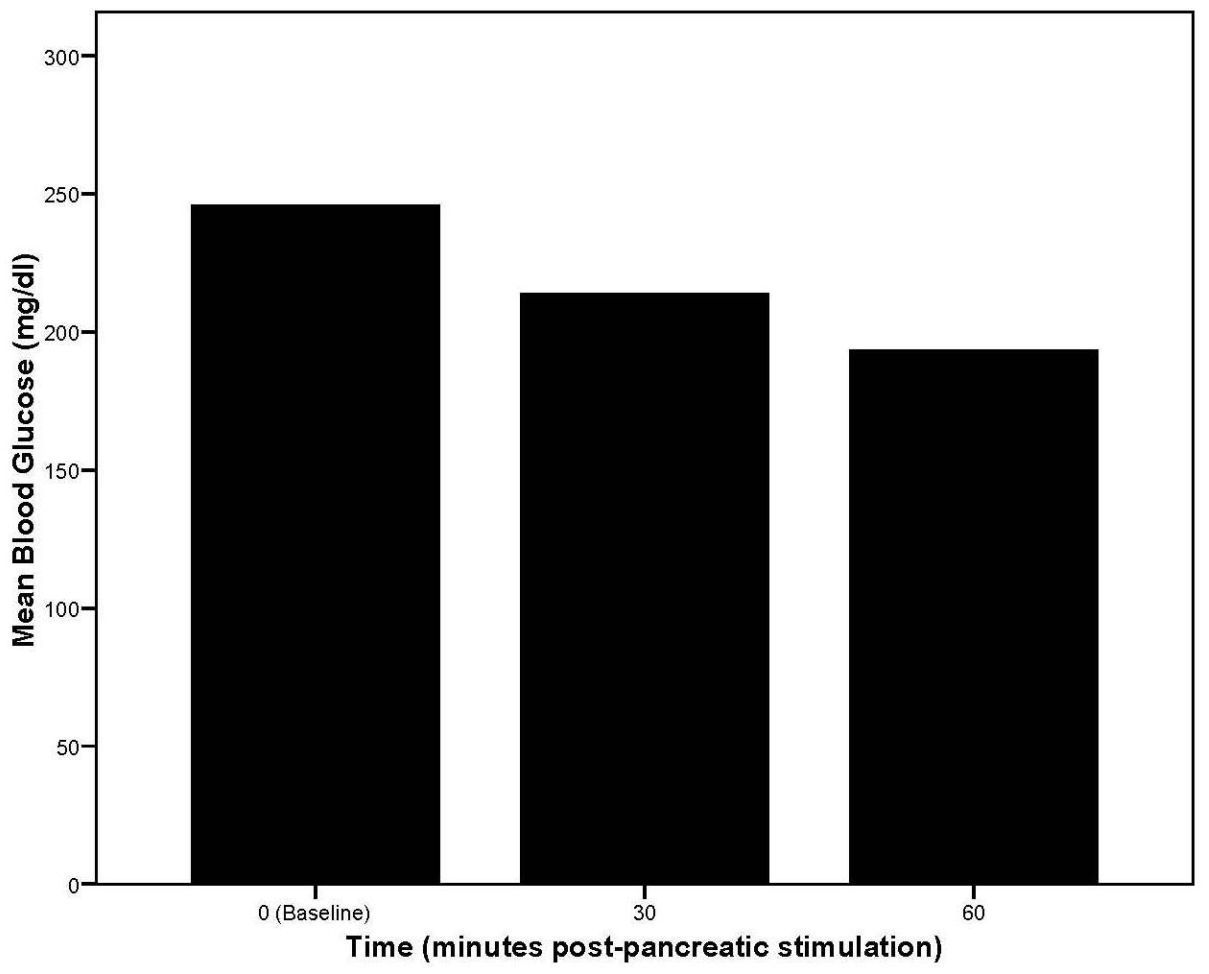
Mean blood glucose levels over time in diabetic patients receiving osteopathic manipulative treatment to stimulate pancreatic functioning. Baseline values are for fasting blood glucose (based on 150, 121, and 86 observations, respectively, at 0, 30, and 60 minutes post-pancreatic stimulation).

A re-analysis of Bandeen's 1949 raw data reveals much about the effects of pancreatic stimulatory and inhibitory OMT techniques in patients with and without diabetes mellitus seen over a 25-year period. The designations of type 1 or type 2 diabetes mellitus were not in use at that time and insulin was the mainstay of diabetes treatment. In the first phase of Bandeen's study, fasting blood glucose was initially measured and patients then received pancreatic stimulation mediated via the lateral chain ganglia in concert with raising of the second, third, fourth, and fifth ribs. Although Bandeen's data table suggests that both diabetic and non-diabetic patients were included in this phase of the study, the lowest fasting blood glucose value was recorded as 110 mg/dl (equivalent to fasting plasma glucose of 126 mg/dl), thereby suggesting that all 150 patients in this phase of the study had diabetes mellitus by contemporary standards [13]. Glucose was then measured 30 (121 patients) and/or 60 (86 patients) minutes following pancreatic stimulation. The results indicated rapid declines in blood glucose in the treated patients as shown in Figure 1. In fact, a case of hypoglycemic coma was recorded as the last observation in this series of patients! Bandeen asserted, "...that islets of Langerhans were not dead but were lying dormant, and relief from diabetic disturbances could be obtained only by relieving the substances keeping them dormant, thereby restoring the normal electrochemical balance of blood protein and tissue colloids [12]."

**Figure 2:**
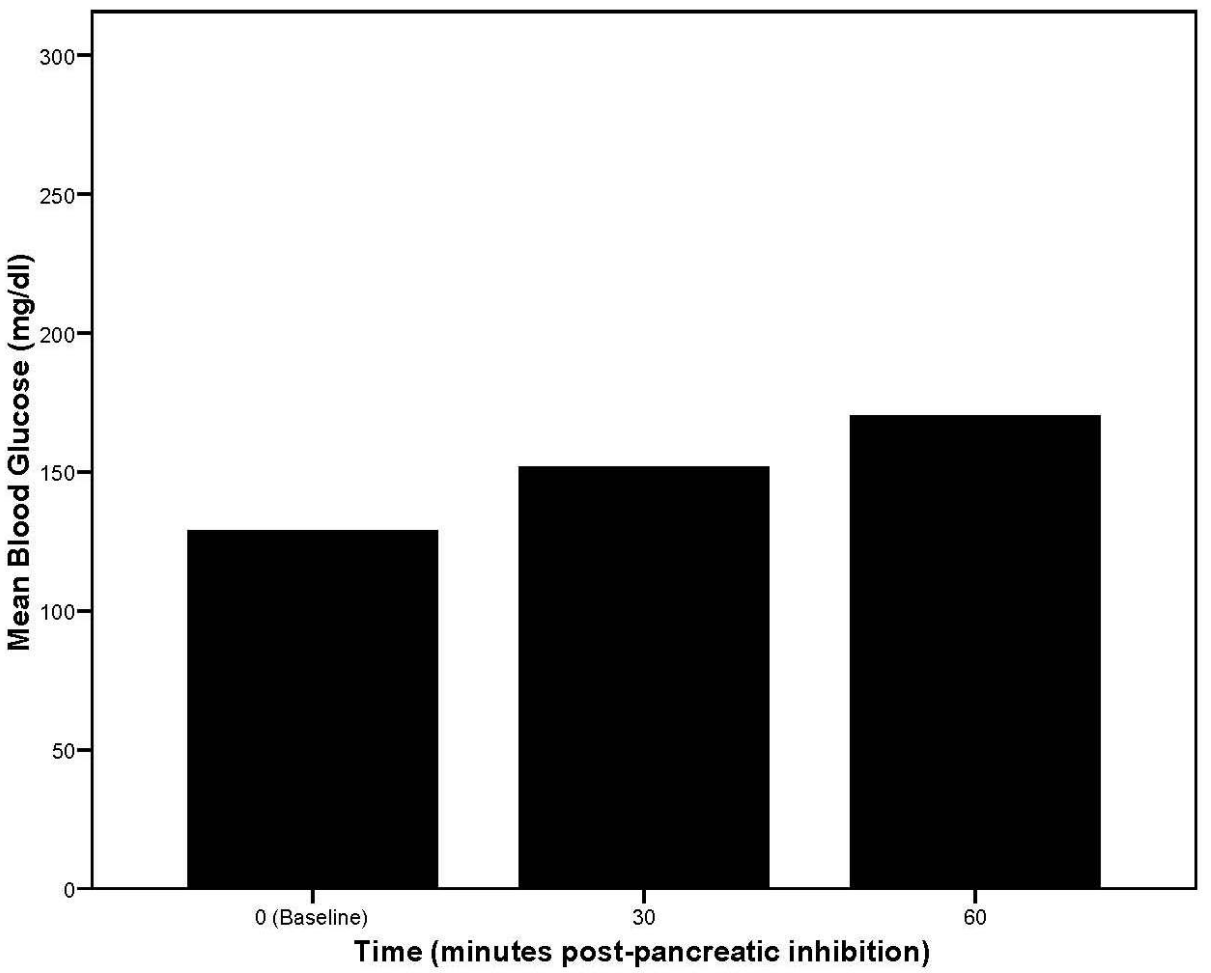
Mean blood glucose levels over time in non-diabetic patients receiving osteopathic manipulative treatment to inhibit pancreatic functioning. Baseline values are for random (non-fasting) blood glucose (based on 40 observations each at 0, 30, and 60 minutes post-pancreatic inhibition).

In the second phase of Bandeen's study, 40 patients were subjected to two minutes of pancreatic inhibition involving specific rotary manipulation of the eleventh and twelfth thoracic and first lumbar vertebrae. Bandeen's data table does not specify that blood glucose measures were performed in a fasting state during this phase of the study. The patients in this phase of the study were likely to be non-diabetic based on the lower baseline (random, non-fasting) blood glucose levels reported, and that the hypothesized effects of pancreatic inhibition would adversely impact blood glucose levels in diabetic patients. Blood glucose was measured before and 30 and 60 minutes after pancreatic inhibition in all 40 patients. The results indicated rapid elevations in blood glucose in the treated patients as shown in Figure 2. Despite methodological shortcomings in reporting of the study design and data by modern standards, and only rudimentary understanding of the mechanisms of diabetes mellitus, these two phases of Bandeen's patient-oriented research provide intriguing results upon which contemporary osteopathic investigators can build.

## Viewing classic osteopathic research through the prism of contemporary medicine

Diabetes mellitus is an important public health problem. Type 2 diabetes mellitus (T2DM) comprises 90% to 95% of the estimated 18.2 million Americans with diabetes mellitus [14]. The prevalence of T2DM in children and adolescents is increasing as a consequence of the continuing rise in obesity in this segment of the population [15]. Early identification of and intervention in patients with T2DM may help control the disease process and delay its complications, including blindness, kidney damage, and lower-limb amputations. Such intervention may also help control the cost of diabetes mellitus, which was estimated to be $132 billion in the United States during 2002, including both medical expenditures and lost productivity [16].

Normal pancreatic β-cells can adapt with a high degree of sensitivity to changes in insulin action [17]. There exists a hyperbolic relationship between β-cell function and insulin sensitivity that continuously degrades with the transition to impaired glucose tolerance and, ultimately, to T2DM. This phenomenon of β-cell dysfunction is a crucial element in the pathogenesis of T2DM which has been verified in longitudinal studies of Pima Indians [18]. Thus, over time, insulin resistance leads to hyperglycemia and T2DM.

Factors strongly associated with insulin resistance include obesity and physical inactivity. The cellular mechanisms that are potentially involved in states of insulin resistance include phosphorylation and dephosphorylation of insulin receptor substrate proteins, inflammatory cytokines released by expanded visceral adipose tissue, reduced concentrations of adiponectin and its insulin-sensitizing effects in liver and muscle, classic inflammatory signaling pathways, and defects in mitochondrial metabolism [19]. Low-grade inflammation appears to be involved in the pathogenetic processes causing T2DM, potentially mediated by tumor necrosis factor-α (TNF-α), interleukin-1β (IL-1β), the IL-6 family of cytokines, and IL-18 [20]. In addition to its immunoregulatory actions, IL-6 has been proposed to affect glucose homeostasis and metabolism directly and indirectly by action on skeletal muscle cells, adipocytes, hepatocytes, pancreatic β-cells, and neuroendocrine cells [20].

Using OMT for the manifestations of T2DM may improve the affected paraspinal tissues and the musculoskeletal system via a generalized non-specific mechanism. Subjects with diabetes mellitus are significantly more likely than non-diabetic subjects to report daily chronic pain; however, their elevated chronic pain risk appears to reflect multiple anatomical locations and is not adequately explained by diabetic neuropathy or obesity [21]. Diabetes mellitus and chronic pain may have common biological factors, such as bradykinin, IL-6, and upregulation of kinin B_1 _or B_2 _receptors. Cytokines acting through kinin B_1 _and B_2 _receptors play a role in the pathogenesis of diabetes mellitus as well as in inflammatory and painful processes [21]. Osteopathic manipulative treatment has been shown to have benefits when used chronically (over 3 to 12 months) in painful low back conditions [22].

In theory, OMT may also effect disruption of a viscerosomatic reflex arc (e.g., a pancreatic viscerosomatic reflex arc) thereby creating the potential for amelioration of the underlying T2DM disease process through a specific mechanism [23]. However, clinical studies to support this theoretical concept are sparse. An important barrier heretofore has been that viscerosomatic reflexes of T2DM have not been empirically demonstrated, but have been based on osteopathic principles. Historically, diabetes mellitus has been viewed as a pancreatic disease, with pancreatic viscerosomatic reflexes expected at the T5-T11 spinal segmental levels [23]. Nevertheless, Bandeen's pancreatic stimulatory OMT technique in diabetic patients may represent a specific OMT technique for treating a pancreatic viscerosomatic reflex arc. In retrospect, Bandeen's specific treatment may have augmented functioning of the pancreatic β-cells by mimicking modern-day drug therapy, potentially via one or more of the following mechanisms: acute stimulation of insulin release, stimulation of insulin biosynthesis, inhibition of β-cell apoptosis, or stimulation of β-cell differentiation [19]. Bandeen also commented on the recuperative power and lasting qualities of pancreatic stimulation, indicating that dramatic improvement in diabetic control could be accomplished within one month and could last up to 15 years in his clinical experience [12].

More recently, a case-control study of osteopathic palpatory findings in T2DM reported significant tissue texture changes at the T11-L2 spinal segmental levels that were suggestive of a renal viscerosomatic reflex arc, and indicative of diabetic nephropathy based on the kidney's autonomic nervous system supply [23]. The argument for causality between underlying renal pathology and the palpated tissue texture changes at T11-L2 was further strengthened by augmented renal viscerosomatic reflex responses observed in T2DM subjects with longer durations of disease (>5 years) and comorbid hypertension. Thus, specific renal stimulatory OMT techniques aimed at the T11-L2 spinal segmental levels may effect disruption of a renal viscerosomatic reflex arc, thereby preventing or delaying the occurrence of diabetic nephropathy in patients with T2DM.

In the current environment that promotes translational research, exciting discoveries involving OMT are being made at several levels. The immediate effects of abdominal lymphatic pump treatment on lymph flow and leukocyte output have been measured using a dog model [24]. Lymphatic pump treatment increased leukocyte counts and lymph flow 2- and 4-fold, respectively, thereby increasing leukocyte flux 8-fold. Enhanced mobilization and lymphatic transport of leukocytes is likely an important mechanism of increased immunity based on these findings. Also, using a dog model, osteopathic palpatory techniques have successfully identified left-sided paraspinal changes at the T2-T5 spinal segmental levels indicative of a viscerosomatic reflex arc attributed to myocardial ischemia [25].

An in vitro model involving human fibroblasts has been developed to simulate and study the effects of indirect OMT techniques on repetitive motion strain [26]. Whereas modeled repetitive motion strain caused a reduction in fibroblast proliferation and a delayed inflammatory response, modeled indirect OMT techniques reversed the inflammatory effects in cells that had been strained repetitively. These findings suggest that fibroblast proliferation and expression and secretion of proinflammatory and anti-inflammatory ILs may contribute to the efficacy of indirect OMT techniques. In another study, human subjects with chronic low back pain had significantly reduced levels of 5-hydroxyindoleacetic acid (a serotonin metabolite) at 30 minutes post-OMT and serotonin at 24 hours post-OMT when compared with baseline concentrations [27].

## Conclusion

Patient-oriented research conducted during the classic era of osteopathy in the United States provides a foundation and, in some cases, useful data for generating hypotheses about the potential mechanisms of action of OMT. Osteopathic investigators would be well-served to rediscover the classic osteopathic literature in their quest to help advance evidence-based medicine.

## Competing interests

The author declares that he has no competing interests.
